# Design, Development and Implementation of the Position Estimator Algorithm for Harmonic Motion on the XY Flexural Mechanism for High Precision Positioning

**DOI:** 10.3390/s20030662

**Published:** 2020-01-24

**Authors:** Mahesh Shewale, Ali Razban, Suhas Deshmukh, Sharad Mulik

**Affiliations:** 1 Department of Mechanical and Energy Engineering, Purdue School of Engineering & Technology, IUPUI, Indianapolis, IN 46202, USA; mshewale@iupui.edu; 2 Department of Mechanical Engineering, Government College of Engineering, Karad, Dist. Satara 415124, India; suhas.deshmukh@gcekarad.ac.in; 3 Department of Mechanical Engineering, RMD Sinhgad School of Engineering, Pune 411058, India; sharadmulik@gmail.com

**Keywords:** flexural, sensorless, micropositioning, estimator, biflex

## Abstract

This article presents a novel concept of the position estimator algorithm for voice coil actuators used in precision scanning applications. Here, a voice coil motor was used as an actuator and a sensor using the position estimator algorithm, which was derived from an electro-mechanical model of a voice coil motor. According to the proposed algorithm, the position of coil relative to the fixed magnet position depends on the current drawn, voltage across coil and motor constant of the voice coil motor. This eliminates the use of a sensor that is an integral part of all feedback control systems. Proposed position estimator was experimentally validated for the voice coil actuator in integration with electro-mechanical modeling of the flexural mechanism. The experimental setup consisted of the flexural mechanism, voice coil actuator, current and voltage monitoring circuitry and its interfacing with PC via a dSPACE DS1104 R&D microcontroller board. Theoretical and experimental results revealed successful implementation of the proposed novel algorithm in the feedback control system with positioning resolution of less than ±5 microns at the scanning speed of more than 5 mm/s. Further, proportional-integral-derivative (PID) control strategy was implemented along with developed algorithm to minimize the error. The position determined by the position estimator algorithm has an accuracy of 99.4% for single direction motion with the experimentally observed position at those instantaneous states.

## 1. Introduction

The progress in the fields of electronics, material science and advanced manufacturing has made increasing demands for ultra-precision technology like micro and nano-positioning stages. These stages have many commercial applications such as laser scanning, biological scanner applications (e.g., tracking of bio cells using optical twizzers), stereolithography applications for the development of prototypes, micromachining and scanning probes (like the scanning tunneling microscope (STM), atomic force microscopy (AFM), etc.) [1] and the use of various actuators like voice coil motors (VCMs) and piezo-electric actuators. The significance of symmetrical topology in XY flexural mechanisms has been a major consideration in reducing errors [2] and this symmetric arrangement of double parallelogram flexural (DPF) modules has been considered in previous work [2,3]. The fundamentals of elastic averaging and its advantages have been illustrated using a three-beam parallelogram flexure mechanism that improves the performance and strength [4]. Symmetric geometry in DPF provides flexibility in design tradeoffs that result in enhanced performance parameters such as positioning accuracy [5]. These mechanisms are especially used in applications like ultra-precision diamond turning machines [6]. In XY positioning, some mechanisms have also been developed using dual servo featuring magnetically levitated motion stage and high accuracy position feedback with capacitive sensors and a laser interferometer [7]. Some synthesized mechanisms like 3-PPR planar parallel manipulators that have a D or U-shaped base have been developed and tested. These also involve compact actuators for the XY stage featuring vibration isolation so that the effect of ground vibration does not impact performance. Furthermore, to enhance the range of motion, multiple DPF mechanisms have been used [8,9,10].

The analytical approach involves coupling between torsion and the bending of flexural elements that have been used in the 3-layer polysilicon micromachining process [11]. Crossed flexural pivots with leaf spring have also been used for a simple design purpose. Shape optimization of these springs offers stress reduction, enhanced fatigue life and an extended range of operation [12]. Micromanipulators based on the flexural mechanism and piezo actuated stages enable an enhanced range of motion. The serial connection of flexural elements was eliminated by the double compound parallelogram system that supports and provides amplification of the motion [13,14]. Different endeavors have been made to use composite materials for flexure design that immensely affect the dynamic response of the system [15]. Rotational motion can also be achieved by using flexural elements, but it results in insufficient damping that puts a constraint on performance [16]. Monolithic mechanism provides ease of manufacturing while eliminating mechanical losses like backlash, wear and friction [17,18,19]. Hyperbolic-shaped, hybrid flexure hinges with monolithic compliant mechanisms are also used in micro electro-mechanical systems (MEMS) devices such as optical shutters and mechanical locks [20]. Efforts have also been made to work on nonlinear behavior of piezo scanners in scanning probe microscopes (SPM) and atomic force microscopy (AFM) [21]. A compliant multi-stable structure was developed using mutually connected tetrahedral units. These compliant, multi-stable structures showed large geometric modification when actuated between their stability regions [22,23,24]. The micromotion XY positioning stage, which was developed by M. Olfania et al. [25], is comprised of electrostatic comb-drive actuators and employs a good motion range. A novel two-stage amplifying mechanism was designed for a large range of motion and high natural frequency ranges. This involves a dynamic model of the positioner from the input/output transfer equation and the parameters obtained from the frequency response analysis [25,26]. The DPF mechanism has been used to design the single DOF flexural mechanism and a novel position estimator algorithm has been implemented to predict the displacement of motion stage. This has eliminated the need for high cost sensors to measure the displacement. Further, proportional-integral-derivative (PID) control was used in this setup to minimize the error between the reference input position and actual displacement of the motion stage [27,28].

A new scanner for AFM was designed for vertical motion, which requires high precision and high bandwidth [29]. The nonlinear characteristics arising from force equilibrium conditions in flexural beams were analyzed to observe the effects of load-stiffening and elasto-kinematic behavior [30]. A magneto-resistive position sensing system was used in the dual-stage nano-positioner, which yields sub-nanometer accuracy over a large work area [31]. A novel XY planar positioning stage was developed by W. Wang et al. for a large motion range without any over-constraints; it eliminates parasitic error and relies on piezo-electric actuators for enhanced accuracy [32]. Electromagnetic actuators can also be used in integration with kinematic and dynamic modeling of mechanical and electromagnetic systems involved. This method involves compliance and stiffness determination using the matrix method [33]. Decoupled prismatic limbs are constructed with compound parallelogram flexures and bridge type displacement amplifiers. This decoupling property was widely adopted in micro and nanoscale manipulators [34,35,36]. High bandwidth nano-positioning systems have also been used in video-rate AFM and probe-based nanofabrication applications. The integral resonant control method was employed in integration with feedforward inversion technique to obtain high speed and accuracy [37,38]. Some precision positioning mechanisms used an inchworm-type piezoelectric actuator that bypasses close tolerance constraints and is easy to assemble [39]. A nano-measuring machine (NMM) was developed using piezo-electric actuators for multipurpose traceable topographic measurements, which require accuracy in nanometers. Exhaustive analysis has been conducted regarding the design methods employed in these mechanisms because of the multiple degrees of freedom required for manufacturing of complicated nanostructures [40,41]. Glenn et al. [6] proposed a method to estimate displacement of voice coil motors for high precision positioning application using sliding mode observer. However, this method is useful only for VCMs that exhibit back-emf that varies with displacement and thus, we needed a different approach to predict the displacement of the motion stage of the mechanism that uses fixed back-emf constant.

Due to the tremendous variety of position sensor techniques and the broad range of applications, it is very challenging to make explicit performance comparisons. In numerous applications, attributes such as the physical size and price play a vital part over performance. Nonetheless, it is illuminating to assess some attributes of performance.

Strain gauges are generally considered to be the simplest and least cost displacement sensor. Due to their size (few mm^2^), strain gauges are appropriate for placing directly on to actuators or stages having a range around 10–500 μm [42]. Though strain gauges can be calibrated to attain high resolution, it is acceptable to consider an error of 1% of the full-scale range (FSR) because of drift and the indirect correlation between the measured strain and real motion. Piezoresistive sensors are tinier than strain gauges and can be attached to actuators that are only 1 mm long with a range of up to 1 m. Though the resolution of piezoresistive sensors is very high, the accuracy is restricted by various factors such as nonlinearity and temperature sensitivity [43]. In the case of piezoresistive sensors, an error limitation of 1% FSR is acceptable. Though strain gauges need contact with actuator or flexural elements, they do not affect any forces between the stationary reference frame and moving platforms, in this sense, they are considered to be of a non-contact type [44].

Capacitive sensors are comparatively simple in construction, offer the greatest value of resolution over small ranges, are oblivious to temperature and can be calibrated to an accuracy of 0.01% FSR [45]. Nevertheless, in common function applications where the sensor is not calibrated after commissioning, alignment errors may restrict the accuracy to 1% FSR [46].

Eddy current sensors can deliver exceptional resolution for motion ranges greater than 100 μm. They are more responsive to temperature than capacitive sensors but are less vulnerable to dust and pollutants, which is essential in industrial atmospheres [47].

Linear variable differential transformers (LVDT) are amongst the highest popular sensors in industrial applications having a range from a few millimeters to tens of centimeters. They are pretty simple and have a high-level inherent linearity. However, they also have a narrow range of operational frequencies and can affect the motion with inertia and friction. The highest resolution is restricted by the physical structure of the transducer, which is usually applicable for ranges greater than 1 mm. The frequency range of operation of LVDT is constrained by the necessity to prevent eddy currents in the core [48].

As compared to other sensor techniques, laser interferometers deliver an extraordinary degree of accuracy. Stabilized interferometers can accomplish accuracy around the range of 1 μm. Nonlinearity is similarly on the order of a few nanometers. Due to the low-noise and intense range of operation, the active range of an interferometer can be very high in the ranges of a few meters [49].

Linear encoders are utilized in similar applications to interferometers where accuracy is the most important parameter. Over larger ranges of operation, accuracy around the range of 1 μm is possible [50]. Even larger accuracies are achievable with linear encoders working on the theory of diffraction. The accuracy of these sensors can exceed 50 nm over ranges of up to 250 mm, which is comparable to the most excellent laser interferometers [51].

All the systems discussed above involve high cost sensors to measure the displacement of the motion stage in the range of a micrometer. The mounting and alignment procedure for these sensors is also tedious and time-consuming. To overcome these issues, we initiated the novel concept of the position estimator algorithm in which the voice coil motor is used as both the actuator and sensor.

This proposed concept and system is applicable in various fields where the voice coil motor is used as an actuator. The idea of physical modeling of the system has not been implemented to date for micro positioning stages having voice coil actuators. Though the method is not novel, the implementation of the idea on a different application proves the novelty and industrial benefits as well. It has numerous applications ranging from meso scale to microscale scanning: laser manufacturing, optical microscopy, optical twizzering, precision metrology equipment and the characterization of micro–nano systems [2,10,14,27]. Furthermore, it can be applicable for biomedical imaging and scanning purposes such as industrial-computed tomography scanning to construct digital 3D models and non-destructive testing. It also has application in the terrestrial laser scanning technique, which is gaining increasing interest due to its advantages of non-contact, high speed, high accuracy and large-scale work envelopes.

## 2. Position Estimator Algorithm

Voice coil actuators for a single stage allow linear movements over a limited range of motion and they were originally used in radio speakers. In speakers, the mass of diaphragm is less, and voice coil actuators are designed to carry designed very small pay load. Voice coil actuators are direct-drive devices based on a permanent magnetic field and current-carrying coil windings, and the actuation force is directly proportional to the applied current. They are used for precision positioning where large range of motion is desired, which is achieved by multiple VCMs in series. These VCMs also provide actuations with zero friction and submicron accuracy.

In a typical closed-loop servo system, the position sensor sends feedback signals to the actuator enabling high speed motion with a high degree of precision and accuracy. Additionally, a position sensor is necessary to provide a feedback loop of the precision position control. In this research, VCM was used for a dual purpose: as an actuator (i.e., its primary objective) and as a sensor to determine the relative position of coil with respect to fixed magnet position. Linear voice coil actuators are available in the range of 1–50 mm with low peak forces varying from 0.7 to 2000 N and strokes that vary from 1 to 50 mm. A typical voice coil actuator manufactured by BEI Kimco Ltd., Vista, CA, USA [27] was used in DPF to apply a desired force on the primary motion stage with required frequency.

### 2.1. Position Estimator Logic

**Figure 1:**
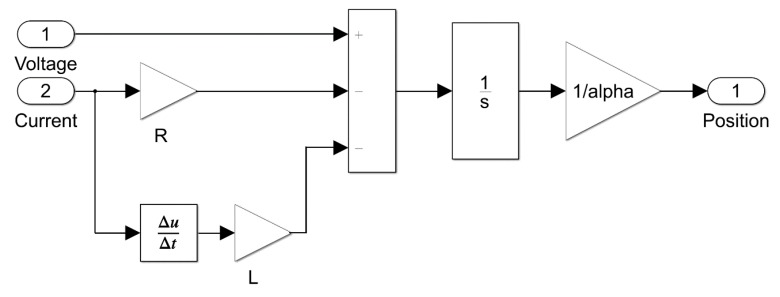
Position estimator algorithm developed in Simulink.

Figure 1 shows a representation of position estimator logic for an electrical model of VCM. The governing equation of electrical circuit voice coil actuator motion is given in (1–3) where $v_{s}\left(t\right)$ is the voltage supplied to VCM in volts, R is the resistance of the coil in ohms, i(t) is the instantaneous current drawn by VCM in amperes, L is the inductance of the coil in milli-Henry, α is the motor constant in N/A, di(t)/dt is the rate of change of current with respect to time and dx(t)/dt is the velocity of the coil.

(1) $$v_{s}\left(t\right)=Ri\left(t\right)+L\frac{di\left(t\right)}{dt}+\alpha \frac{dx\left(t\right)}{dt}.$$

(2) $$\frac{dx\left(t\right)}{dt}=\frac{1}{\alpha }\left(v_{s}\left(t\right)-Ri\left(t\right)-L\frac{di\left(t\right)}{dt}\right).$$

(3) $$x\left(t\right)=\frac{1}{\alpha }\int \left(v_{s}\left(t\right)-Ri\left(t\right)-L\frac{di\left(t\right)}{dt}\right)dt.$$

Using Equation (3), we developed an algorithm for the measurement of the stroke of the voice coil actuator with zero initial conditions as shown in Figure 1. Here, the instantaneous value of the current is a result of Ohm’s law using instantaneous voltage supplied to VCM and the value of the resistance of the coil. The instantaneous voltage was measured in real time across the two terminals of the voice coil.

### 2.2. MATLAB Simulation of Position Estimator

**Figure 2:**
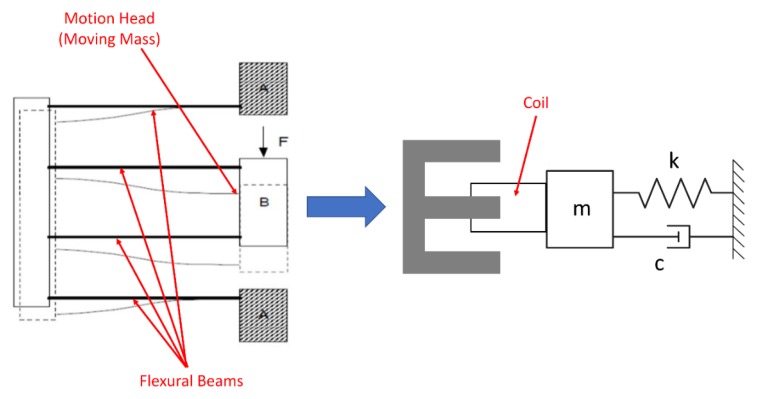
Representation of the double parallelogram flexural (DPF) with the voice coil motor (VCM) in an electromechanical model.

Position estimator algorithm presented in Section 2.1, was simulated using MATLAB SIMULINK software with DPF mechanism as single DOF mechanism. Figure 2 shows a representation of DPF with VCM equivalently in electrical and mechanical model. Part A denotes a fully constrained fixed block on which the permanent magnet of VCM was mounted. Part B shows moving mass on which a coil of VCM was mounted. This flexural model was equivalently considered as a single DOF spring mass damper system in which m is the moving mass, k is the stiffness and c is the damping coefficient. Properties of the mechanical and electrical systems are tabulated in Table 1.

### 2.3. Simulink Model

**Figure 3:**
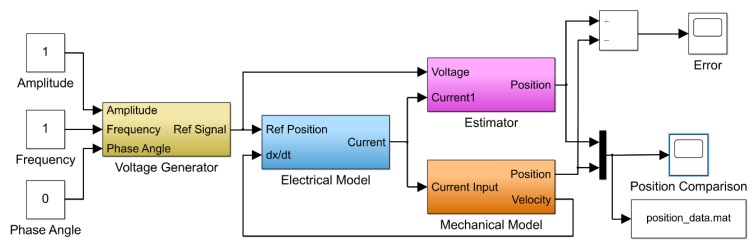
Simulink model for the position estimator algorithm.

The Simulink model containing a voltage generator, electrical model, mechanical model and position estimation model is shown in Figure 3. The estimator sub-model consisted of mathematical modeling using Equation (3). Input for current and voltage in the simulation was the pure sine wave function based on desired amplitude and frequency generated by the voltage generator block.

**Figure 4:**
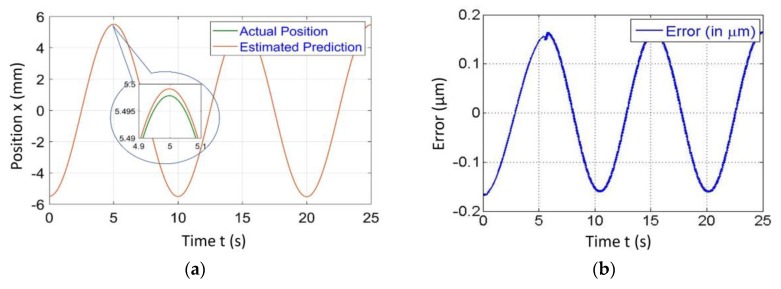
Simulated results of the position estimator algorithm at 0.1 Hz frequency for (**a**) Position comparison and (**b**) Error between actual and estimated position.

**Figure 5:**
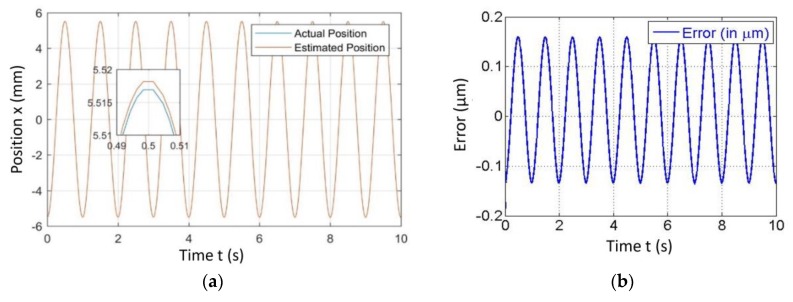
Simulated results of the position estimator algorithm at 1 Hz frequency for (**a**) Position comparison and (**b**) Error between actual and estimated position.

**Figure 6:**
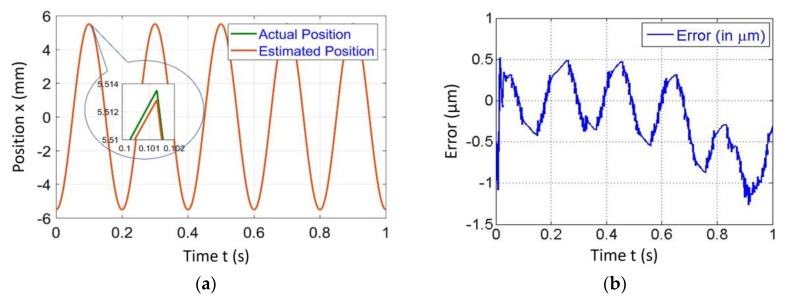
Simulated results of the position estimator algorithm at 5 Hz frequency for (**a**) Position comparison and (**b**) Error between actual and estimated position.

**Figure 7:**
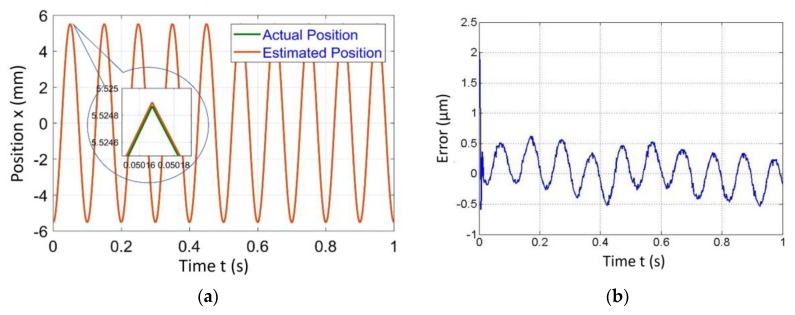
Simulated results of the position estimator algorithm at 10 Hz frequency for (**a**) Position comparison and (**b**) Error between actual and estimated position.

Figure 4, Figure 5, Figure 6 and Figure 7 represent a comparison of the position estimator output and actual position from mechanical simulation at frequencies of 0.1, 1, 5 and 10 Hz. In each figure, “(a)” represents the comparison of estimator and actual, and “(b)” is the error between those values. The summary of comparison error at various frequencies is given in Table 2. The error range was ±0.14 to ±0.6 µm.

Figure 3 illustrates the mechanical model, electrical model, position estimation model and voltage generator. Voltage input was provided to the electrical model via the voltage generator, which generates the current signal and was further fed to the mechanical model. The mechanical model gave the output as the displacement and velocity. Stroke estimation block was used to estimate a stroke value based on current and voltage signal. Further, estimated stroke and stroke (position) obtained from the mechanical model were compared.

The errors shown in the Figure 4, Figure 5, Figure 6 and Figure 7 were peak to peak values, but the values of error mentioned in the related text and Table 2 were only a one side error since both input and output were the sine wave. The need to test the algorithm for various frequencies comes into picture later in the case of 2-D scanning. In this case, there is a need of different operating frequencies to track various paths in 2D plane. As a reason, amplitude was kept constant and frequency was varied. The effect of ground vibrations increased and came into the picture as we increased frequency, which could be seen in Figure 6 and Figure 7. In this case, the asymmetric behavior of error was due to the transient behavior of mechanical and electrical models involved.

## 3. Development of the Experimental Setup

**Figure 8:**
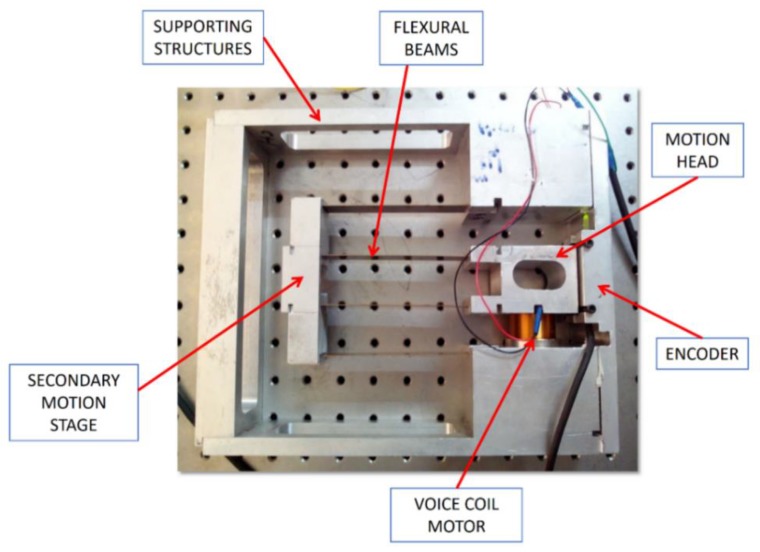
Manufactured DPF manipulator.

For validation, we developed a flexural mechanism based on a double flexural mechanism to eliminate the parasitic error and angular rotation error as shown in Figure 8. Here, the parasitic error refers to the unwanted displacement of motion stage in the direction perpendicular to the force applied. Investigation describes that DPF yields no parasitic error displacement and results in perfect straight-line motion. Displacement (δ in mm) of a DPF was obtained using Equation (4) [28] in which *F* is the force applied by VCM in N; *L* is the length of the flexural beam in mm; *E* is the modulus of elasticity for beam material in N/mm^2^, *I* is the area moment of inertia in mm^4^, *b* is the width of the beam in mm and *d* is the height of the beam in mm.

(4) $$\delta =\frac{FL^{3}}{12EI}\;\mathrm{and}\;I=\frac{bd^{3}}{12}.$$

The range of parameters selected for the mechanism was based on the linearity of the material selected (i.e., beryllium copper). A required deflection of ±5 mm was considered for our model as the geometrical constraint of the design. The required force was 5 N and it was determined based on the linear behavior of material used for the mechanism. Ranges of length, width and thickness, which were selected based on Equation (4), are given in Table 3.

The DPF model was analyzed for the linear operating range with a constant exciting force of 5 N. The beams used are cantilever and mass were attached at the free end. It was observed from a parametric analysis that the required deflection was obtained with minimum stresses when length = 100 mm, width = 20 mm and thickness = 0.5 mm. The calculated area moment of inertia, deflection, damping factor and theoretical stiffness were I = 270.83 × 10^−3^ mm^4^, δ = 7.91 mm, ζ = 0.00125 and k = 0.6321 N/mm respectively. Using these dimensions, a single DOF positioning mechanism was developed using DPF as shown in Figure 8.

### Biflex Mechanism

**Figure 9:**
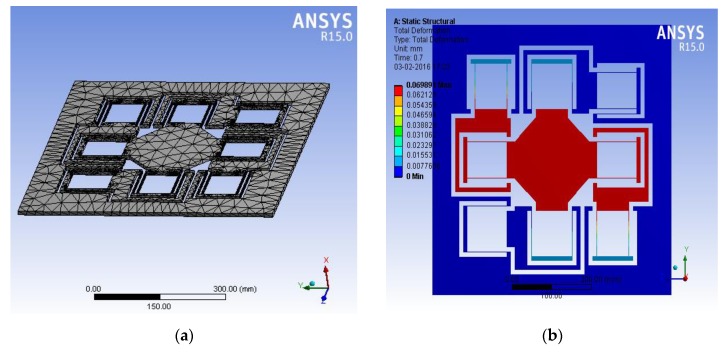
ANSYS simulation of the biflex mechanism.

Planar flexural mechanism was analyzed using the feature-based parametric modeling technique finite element analysis (FEA) tool ANSYS as shown in Figure 9 below. Figure 9a shows meshing with 28,497 nodes and 13,510 quad elements. Figure 9b shows displacement of the biflex mechanism in the X direction only. The objective was to find dimensions of the flexural beam that give the desired range with minimum stresses being developed.

**Figure 10:**
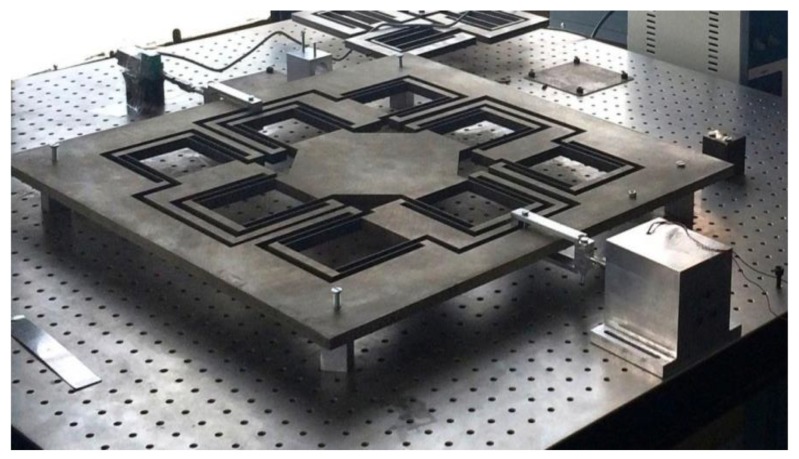
Manufactured XY flexural mechanism.

FEA was carried out to analyze variation in length, thickness and width of flexural beam of the DPF-based biflex model. The dimensions of flexural beams were finalized the same as that of DPF. This biflex mechanism was manufactured using a standard wire electrical discharge machining (EDM) process. Figure 10 shows a manufactured XY flexural mechanism. The material used for the mechanism was stainless steel.

## 4. System Identification

**Figure 11:**
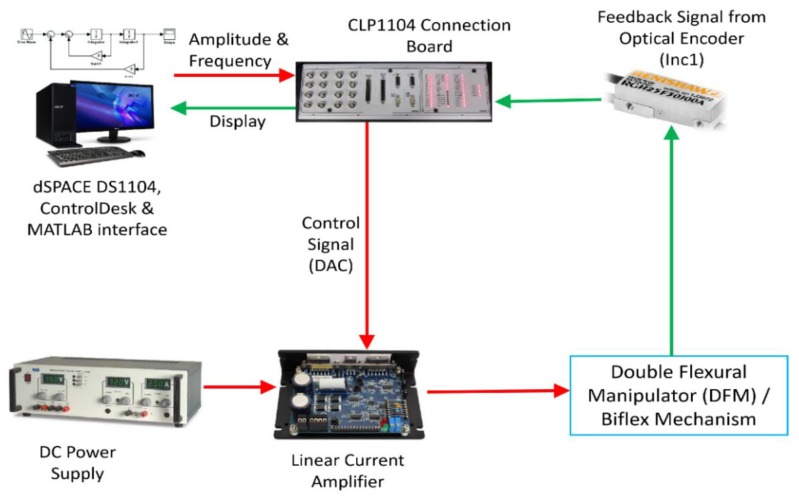
Mechatronic integration for the biflex mechanism.

The developed biflex mechanism was interfaced with PC for further experimental analysis and testing. Mechatronic integration involved connecting/communicating the mechanical system with PC via various electronic and microcontroller systems. It contained the mechanical system, sensors, actuators, a power amplifier, microcontrollers and interfacing software. Once assembly and alignment were ensured with appropriate accuracy, a flexural mechanism was further interfaced with a PC-computer by mounting an optical encoder via a microcontroller. Figure 11 shows a system integration, which connects the XY mechanism, VCM, Renishaw RGH22 Optical encoder to the dSPACE DS1104 Controller.

### 4.1. Static Analysis

**Figure 12:**
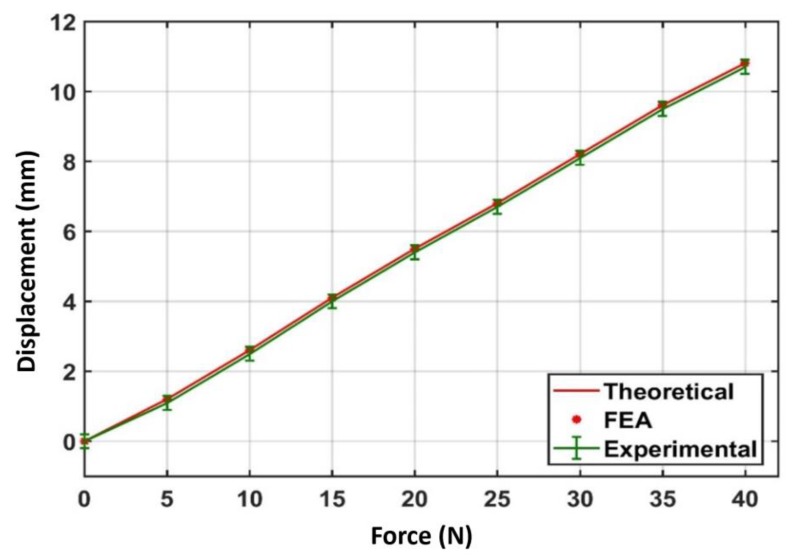
Comparison of theoretical, finite element analysis (FEA) and experimental force deflection characteristics.

Figure 12 shows a comparison between theoretical, simulation using FEA and experimental results. It was observed that force–deflection curve was linear and had a fixed slope. The slope of this line represents the stiffness of the flexural mechanism. The experimental force–deflection curve was further compared with FEA results and it was observed that there was very good agreement of around ±50 microns.

### 4.2. Dynamic Analysis

Before determining transient response of the system, it was necessary to estimate the natural frequency of the system. The mass of all of these components was measured using an electronic balance machine; it was 6.335 kg and stiffness was K=3.688 N/mm. The undamped natural frequency of the system was calculated to be $\omega_{n}=24.12\;\mathrm{rad}/s=3.84\;\mathrm{Hz}.$ System response for the step input was determined to show the behavior of the system towards changing input conditions.

Logarithmic decrement $\delta_{1}$ was used for the calculation of damping factor, which is given by Equation (5) in which *X*_0_ and *X_n_* are the consecutive peak values between n oscillations obtained in step response.

(5) $$\delta_{1}=\frac{1}{n}\left[log\left(\frac{X_{0}}{X_{n}}\right)\right]=0.0809.$$

Damping factor (ζ) is given by Equation (6),

(6) $$\zeta =\frac{\delta_{1}}{\sqrt{4\pi^{2}+\delta_{1}}}=0.0128.$$

**Figure 13:**
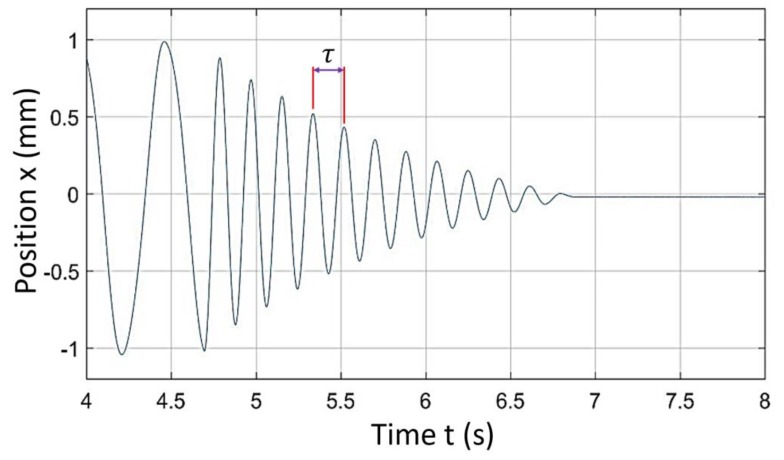
Comparison of theoretical, FEA and experimental force deflection.

The damped natural frequency was $\omega_{d}=\frac{1}{\tau }=\frac{1}{0.288}=3.472\;\mathrm{Hz}$, where τ is the time constant measured between two consecutive peaks as shown in Figure 13.

Figure 13 shows a step response of a current system. It clearly had significant damping of vibrations due to system structural damping. From the step response, both the damping factor (*ζ*) and the damped natural frequency (*ω_n_*) were determined. From these values of *ζ* and *ω_n_*, we determined transfer function for X direction as given in Equation (7).

(7) $$G\left(s\right)=K\frac{\omega_{n}^{2}}{s^{2}+2\zeta \omega_{n}s+\omega_{n}^{2}}=\frac{1.0136}{s^{2}+3.458s+580.13}.$$

**Figure 14:**
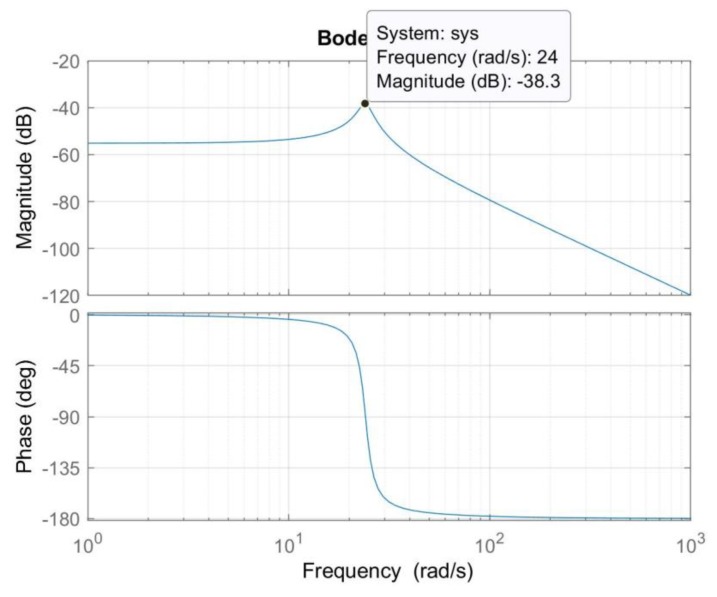
Experimental frequency response.

The frequency response for the transfer function is given in Figure 14. From the frequency response, we found that experimental natural frequency was 24 rad/s, which was close to the theoretical natural frequency of 24.12 rad/s at the beginning of this section.

## 5. Experimental Validation of the Position Estimator Algorithm

**Figure 15:**
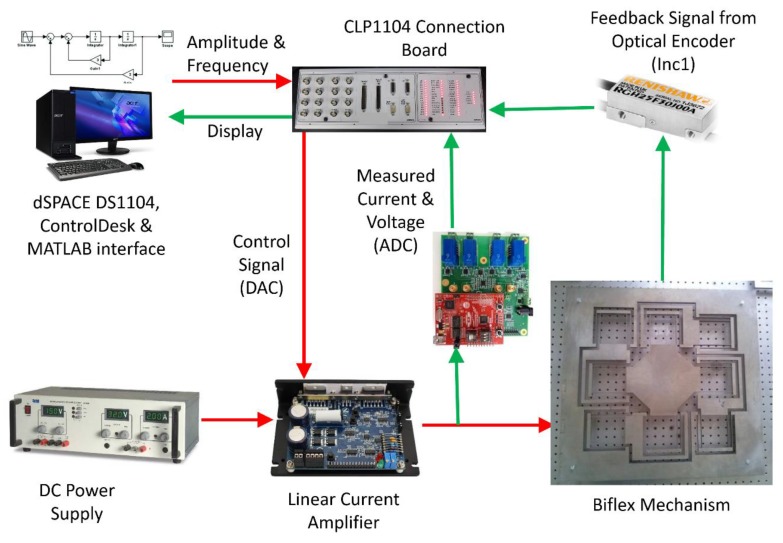
Experimental setup for sensorless validation.

Figure 15 shows a mechatronic integration of the sensorless operation on a given mechanism, which uses VCM as an actuator. It consisted of a mechanism mounted with VCM, LCAM (linear current amplifier), DC power supply, DS1104 microcontroller with CLP board, PC equipped with MATLAB software, RTI, RTW, control desk environment optical encoder and current and voltage monitoring circuitry. PC-generated command signal through a control desk graphical user interface (GUI) environment and ds1104 microcontroller converted digital signal to analogue signal via DAC port. Furthermore, the DAC port was connected to LCAM, which amplifies the signal and drives the voice coil motor with a commanded signal. The VCM-generated linear force and coil of VCM was rigidly fixed to the motion stage of the mechanism. Displacement of the motion stage was dependent on the direction of the current. During displacement of the motion stage, the voltage and current monitory circuit continuously monitored the voltage and current drawn by the voice coil motor. A coil of VCM was rigidly fixed, voltage and current drawn by the coil were further processed as presented in the algorithm, and relative position of the motion stage was predicted.

**Figure 16:**
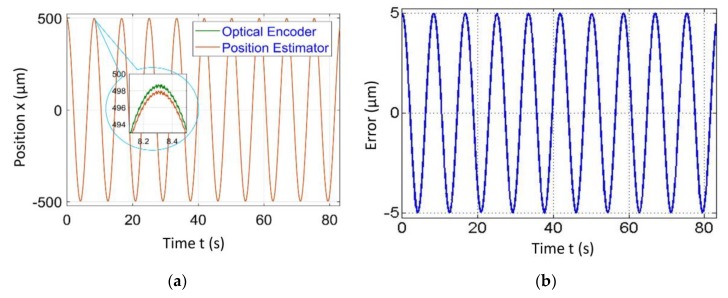
Position estimator output for amplitude = 500 µm and frequency = 0.75 Hz at a scanning speed = 1.5 mm/s.

**Figure 17:**
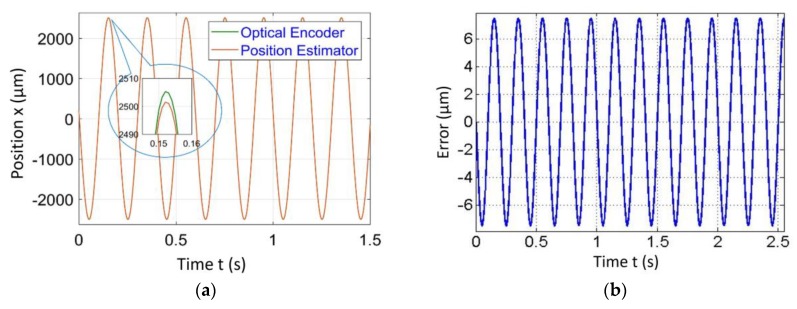
Position estimator output for amplitude = 2500 µm and frequency = 5 Hz at a scanning speed = 50 mm/s.

The procedure described above was adopted for the demonstration of the proposed position estimator algorithm. Hence, the input signal was commanded to VCM and the output amplitude was measured in two ways, one by position estimator and one by an optical encoder at various frequencies. The Renishaw RGH22 optical encoder having a resolution of 50 nm was used solely for validation of the position estimator algorithm. It was observed that there was a close match between both results and error in the position estimator, which gives sub µm accuracy in the range of 2–5 µm for the amplitude ranging from 500 to 2500 µm respectively. The results of the position estimator algorithm for amplitudes ranging from 500 to 2500 µm, frequencies ranging from 0.75 to 5 Hz at speeds of 1.5 and 50 mm/s are given in Figure 16 and Figure 17. It was observed that the difference between position estimator output results and actual optical encoder output were ±5 to ±7 µm.

## 6. PID Control Implementation

**Figure 18:**
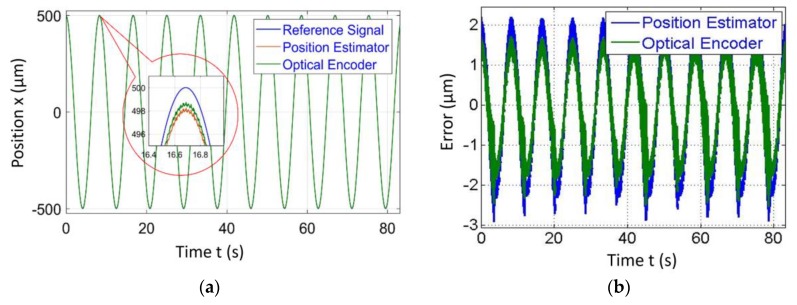
Position estimator output for amplitude = 500 µm and frequency = 0.15 Hz at a scanning speed = 1.5 mm/s.

**Figure 19:**
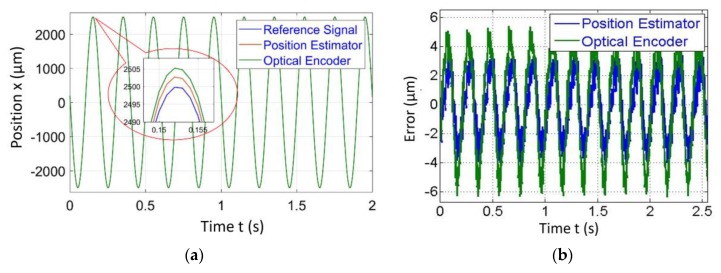
Position estimator output for amplitude = 2500 µm and frequency = 5 Hz at a scanning speed = 50 mm/s.

Figure 18 and Figure 19 demonstrate the PID control implementation results on the biflex mechanism at various amplitudes and frequencies. We observed the difference of ±3 µm and ±6 µm at scanning speeds of 1.5 mm/s and 50 mm/s respectively.

**Figure 20:**
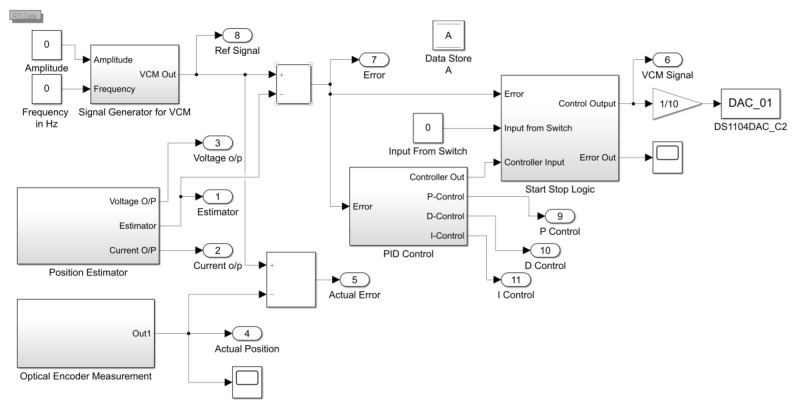
Proportional Integral Derivative (PID) control integration with position estimator algorithm.

PID control logic was used in the integration with the position estimator algorithm to achieve better accuracy as shown in Figure 20. It calculated the error between the reference position and predicted position, and it generated actuation signals to send to VCM via DAC port. In this research we got two sensor feedback outputs. One from the proposed novel position estimator algorithm that generated a position output signal as described in previous sections and another from the optical encoder, which was only used for validation. Considering the algorithm output as a feedback, we implemented the PID controller as shown in Figure 20 and PID parameters were tuned using the standard Ziegler–Nichols scheme.

## 7. XY Scanning Using the Position Estimator Algorithm

The developed novel algorithm was initially implemented on a single DOF flexural mechanism. Further, it was implemented on the biflex mechanism for a more application-oriented validation purpose.

**Figure 21:**
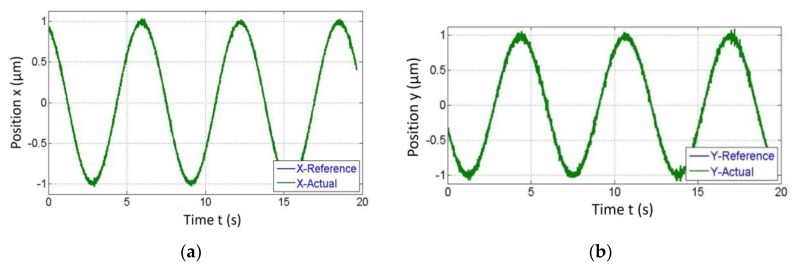
Separate outputs from the algorithm in each the (**a**) X and (**b**) Y direction for tracking a circle of diameter 2mm.

**Figure 22:**
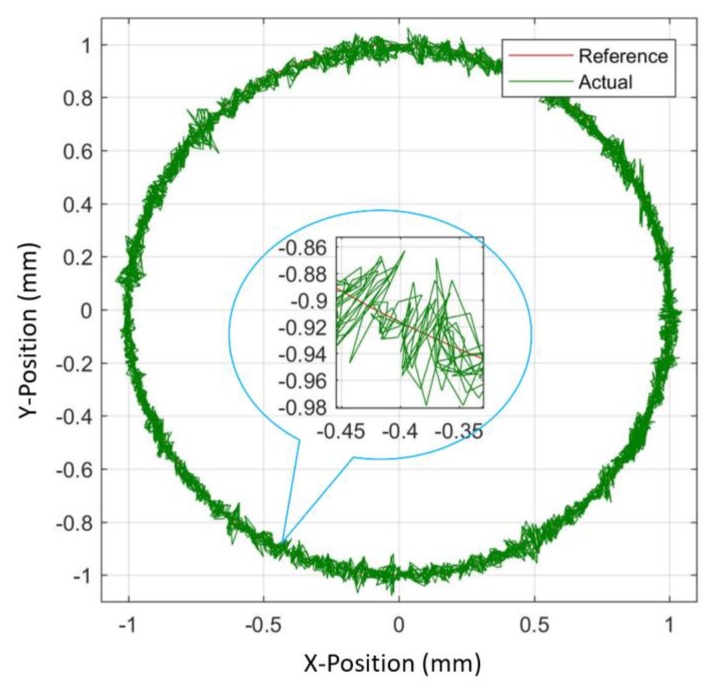
Experimental demonstration of the position estimator algorithm for tracking a circle with a diameter of 2 mm.

A reference circle of diameter 2 mm was resulted as an outcome of the same amplitude = 1 mm and frequency = 0.15 Hz provided to both X and Y direction VCMs. In this case, 2D tracking results from the measurement of two different signals each measure in X and Y directions as shown in Figure 21a,b. These separate signals were then plotted on the XY plot to display tracking in the 2D plane as shown in Figure 22. To trace a circle of diameter 2 mm, two sine inputs were given at a phase difference of 90° and the mentioned of 99.4% was the accuracy for these plots.

**Figure 23:**
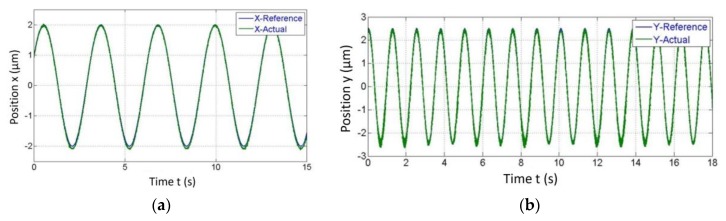
Separate outputs from the algorithm in each the (**a**) X and (**b**) Y direction for tracking a zigzag path.

**Figure 24:**
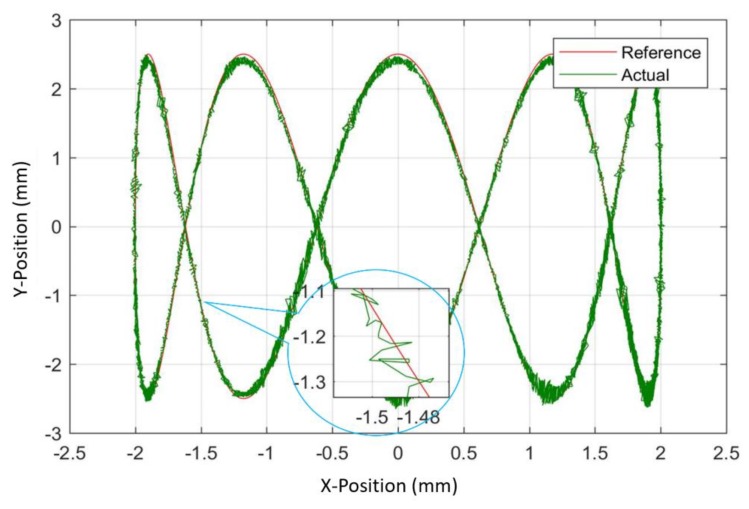
Experimental demonstration for tracking a Lissajous path.

We found the accuracy of around 5–10 µm between the reference input position and position predicted by the developed algorithm. The same input strategy was adopted for the zigzag path as that of the circle. The only change done was the frequency of the Y-direction actuator was increased from 0.15 to 0.75 Hz as shown in Figure 23b and it yielded in zigzag tracking of the motion stage. Figure 24 shows the tracking of motion stage in the Lissajous way close agreement of around 99.2% between the optical encoder output and estimator algorithm output was observed. Similarly, any two-dimensional path could be scanned by providing appropriate amplitude and frequency to the X and Y direction VCMs. 

## 8. Conclusions

Design of the DPF, biflex mechanisms and their experimental setup were developed. The mechatronic integration was carried out using dSPACE DS1104 microcontroller. System identification was carried out and experimental model was developed. Developed transfer function was validated experimentally. Furthermore, novel position estimator algorithm was developed for flexural based scanning system using VCM as an actuator and the accuracy of 99.4% was achieved for single direction and 99.2% for 2D scanning. The PID control feedback was used for the proposed novel position estimator and it was validated with due experimentation. It makes sense to say that error is proportional to the displacement of the motion stage at higher frequencies. It was observed that the positioning resolution was ±2.5 µm at a speed of 1.5 mm/s scanning. Position resolution and accuracy achieved using the PID control were summarized in Table 4.

The proposed novel position estimator algorithm eliminated the use of the current high cost sensors for precise positioning. This will further eliminate sensor alignment; mounting difficulties and it would result in overall cost reduction of the scanning mechanism. This algorithm has a wide range of applications that range from micro scanning for micro manufacturing to any microscopic applications. Further, this algorithm can be tested for various types of inputs such as triangular or square wave input and system disturbances as well.

## Figures and Tables

**Table 1:** Properties of the mechanical and electrical system.

Sr. No.	Parameter	Value
1	Stiffness (k)	0.63 N/mm
2	Damping Coefficient (c)	0.0345 Ns/mm
3	Mass (m)	11.7 × 10^−3^ kg
4	Resistance (R)	9.1 Ω
5	Inductance (L)	4.2 mH
6	Force Sensitivity (α)	8.81 N/A

**Table 2:** Summary of error at various frequencies.

Amplitude (mm)	Frequency (Hz)	Error (µm)
5.5	0.1	±0.14
	1	±0.16
	5	±0.4
	10	±0.6

**Table 3:** Ranges of dimensions for parametric analysis.

Parameter of Beam	Range in mm
Length	50–150
Width	10–30
Thickness	0.5–1

**Table 4:** Summary of achievements of the position estimator algorithm.

Parameter	Sensor Used for Validation	Position Estimator Output	
		Without PID	With PID
Scanning range	15 mm	15 mm	15 mm
Position Resolution	±2 μm	±5 μm @1.5 mm/s	±2.5 μm @1.5 mm/s
